# Glucose and Glycogen Metabolism in *Brugia malayi* Is Associated with *Wolbachia* Symbiont Fitness

**DOI:** 10.1371/journal.pone.0153812

**Published:** 2016-04-14

**Authors:** Denis Voronin, Saheed Bachu, Michael Shlossman, Thomas R. Unnasch, Elodie Ghedin, Sara Lustigman

**Affiliations:** 1 Molecular Parasitology, New York Blood Center, Lindsley F. Kimball Research Institute, New York, NY 10065, United States of America; 2 Global Health Infectious Disease Research Program, Department of Global Health, College of Public Health, University of South Florida, Tampa, FL 33612, United States of America; 3 Department of Biology, Center for Genomics & Systems Biology, and College of Global Public Health, New York University, New York, NY 10003, United States of America; New England Biolabs, UNITED STATES

## Abstract

*Wolbachi*a are endosymbiotic bacteria found in the majority of arthropods and filarial nematodes of medical and veterinary importance. They have evolved a wide range of symbiotic associations. In filarial nematodes that cause human lymphatic filariasis (*Wuchereria bancrofti*, *Brugia malayi*) or onchocerciasis (*Onchocerca volvulus*), *Wolbachia* are important for parasite development, reproduction and survival. The symbiotic bacteria rely in part on nutrients and energy sources provided by the host. Genomic analyses suggest that the strain of *Wolbachia* found in *B*. *malayi* (*w*Bm) lacks the genes for two glycolytic enzymes—6-phosphofructokinase and pyruvate kinase—and is thus potentially unable to convert glucose into pyruvate, an important substrate for energy generation. The *Wolbachia* surface protein, wBm00432, is complexed to six *B*. *malayi* glycolytic enzymes, including aldolase. In this study we characterized two *B*. *malayi* aldolase isozymes and found that their expression is dependent on *Wolbachia* fitness and number. We confirmed by immuno-transmission electron microscopy that aldolase is associated with the *Wolbachia* surface. RNAi experiments suggested that aldolase-2 plays a significant role in both *Wolbachia* survival and embryogenesis in *B*. *malayi*. Treatment with doxycycline reduced *Wolbachia* fitness and increased the amount of both glucose and glycogen detected in the filarial parasite, indicating that glucose metabolism and glycogen storage in *B*. *malayi* are associated with *Wolbachia* fitness. This metabolic co-dependency between *Wolbachia* and its filarial nematode indicates that glycolysis could be a shared metabolic pathway between the bacteria and *B*. *malayi*, and thus a potential new target for anti-filarial therapy.

## Introduction

Endosymbiotic, maternally transmitted bacteria of the genus *Wolbachia* (family *Rickettsiaceae*) reside in the cytoplasm of all reproductive and some somatic cells of the majority of arthropods and in most of the filarial nematodes of medical and veterinary importance, including *Wuchereria bancrofti*, *Brugia malayi*, *Onchocerca volvulus*, *O*. *ochengi* and *Dirofilaria immitis* [1,2]. *Wolbachia* and their hosts have evolved an array of symbiotic associations, ranging from reproductive parasitism in arthropods, to mutualistic relationships observed in wasps and filarial nematodes [1,3,4]. Human filarial parasites infect people in more than 73 countries worldwide, causing debilitating chronic diseases such as lymphatic filariasis (elephantiasis) and onchocerciasis (river blindness) [5]. Elimination of *Wolbachia* from filarial nematodes by treatment with antibiotics affects parasite development, reproduction, and survival, confirming the obligatory role of the bacteria for worm fitness and development [2,5–8]. Clinical trials testing the effects of therapy with antibiotic drugs, such as doxycycline or rifampicin, demonstrate that clearance of *Wolbachia* in the filaria results in slow death of the worms, preceded by sterilization and reduction in transmission [7,9–11]. Doxycycline is recommended as a treatment of choice in certain situations [12]. However, the use of the antibiotic for mass drug administration, a measure currently used to control transmission of these infections, is not practical because of the need for long-term treatment (4–6 weeks) and the contraindication for young children (<8 years of age) and lactating or pregnant women.

As the underlying molecular basis for the *Brugia malayi-Wolbachia* co-dependency remains largely unknown, elucidating the specific processes involved could reveal promising new anti-filarial drug targets. Genomic analyses have highlighted molecular gaps in the metabolic pathways of both *Wolbachia* and *B*. *malayi*, suggesting that there may be some functional complementation occurring. For example, the filarial nematode *B*. *malayi* lacks the genes necessary to produce flavin adenine dinucleotide (FAD), riboflavin, and heme; conversely the *Wolbachia* of *B*. *malayi* (*w*Bm) cannot synthesize *de novo* biotin, nicotinamide adenine dinucleotide (NAD), Co-A, ubiquinone and folate [13–16]. Conversely, the *w*Bm genome does not encode the genes for two glycolytic enzymes (6-phosphofructokinase and pyruvate kinase) that are necessary to convert glucose into pyruvate [17–19], while *B*. *malayi* has the genes for a fully functional glycolytic pathway. However, *w*Bm has the complete machinery necessary to utilize pyruvate in gluconeogenesis and for energy generation via the TCA cycle. Possible compensatory biosynthetic pathways and shared metabolic pathways could account for much of the obligatory co-dependency between the bacteria and its filarial host.

Recently, we demonstrated that the *w*Bm surface protein wBm00432 is associated with six *B*. *malayi* glycolytic enzymes: fructose-1,6-bisphosphate aldolase, triosephosphate isomerase, L-lactate dehydrogenase, enolase, glyceraldehyde-3-phosphate dehydrogenase, and phosphoglycerate kinase [19]. We postulated that the ability of *w*Bm to sequester the host glycolytic enzymes onto their surface could facilitate the localized breakdown of glucose into pyruvate. The pyruvate, once transported into the bacterial cell, might be used in gluconeogenesis and/or enter the TCA cycle, resulting in glucose and energy production in the bacterial cell. Therefore, *w*Bm could depend on the products produced by the *B*. *malayi* glycolytic pathway. Here, we hypothesize that *Wolbachia* might have evolved the ability to regulate the intracellular amounts of glucose and glycogen, which are the substrates for the host’s glycolytic pathway. This would make glycolytic enzymes, such as aldolase, an important physiological bridge between the parasite and its endosymbiont. Here, we characterize the two isozymes of fructose-1,6-bisphosphate aldolase encoded in the *B*. *malayi* genome (BMA-ALDO-1 and BMA-ALDO-2) and describe a unique role that BMA-ALDO-2 may play in symbiosis.

## Materials and Methods

### Parasite material and antibiotic treatment

Adult *Acanthocheilenema viteae* parasites were provided by the NIH/NIAID Filariasis Research Reagent Resource Center (FR3), University of Wisconsin, Oshkosh. Parasites were recovered from subcutaneously infected Golden Syrian LVG Hamsters. *B*. *malayi* parasites recovered from the peritoneal cavity of infected gerbils (*Meriones unguiculatus*) were also obtained from FR3, University of Georgia Athens. All animal work conducted by FR3 followed the national and international guidelines outlined by the National Institutes of Health Office of Laboratory Animal Welfare, and was approved by the University of Georgia Athens and the University of Wisconsin Oshkosh Institutional Animal Care and Use Committees under protocol numbers A2013 11–009 and 0026-000229-R1-04-25-13, respectively. Microfilariae (Mf, *B*. *malayi*) and adult worms (*B*. *malayi* and *A*. *viteae*) were cultured in 5 ml of complete culture medium (RPMI-1640 supplemented with 10% FBS, 100 U/mL penicillin, 100 mg/mL streptomycin, 2 mM L-glutamine, 2.5 mg/mL amphotericin B, and 25 mM HEPES (GIBCO)) at 37°C under 5% CO_2_. In all experiments, parasites (*B*. *malayi* and *A*. *viteae*) were cultured first overnight to confirm their fitness before subjecting them to various treatments. Mf (*B*. *malayi*) and adult worms (*B*. *malayi* and *A*. *viteae*) were treated *in vitro* with 12.5 μM doxycycline in complete medium following previously published protocols [20,21]. Treatment was carried out for 3 or 6 days. Control worms were cultured in complete medium containing an equal volume of DMSO present in the antibiotic treated wells.

### RNA isolation and production of cDNA, dsRNA and siRNA

Total RNA was extracted from untreated *B*. *malayi* adult female worms using a TRIzol-based method and PureLink^®^ RNA Mini Kits with on-column DNase I treatment (Ambion), as described previously [22]. This RNA (2.5 μg) was used as a template for cDNA synthesis using the SuperScript III First Strand cDNA Synthesis Kit (Invitrogen). Standard PCR was performed using cDNA and gene-specific primers flanked by T7 promoter sequences (Table 1). The PCR products (a single product for each set of primers) were then gel-extracted and cloned using the TOPO^®^ TA Cloning^®^ Kit with One Shot^®^ TOP10 Chemically Competent *E*. *coli* (Invitrogen). To obtain templates for dsRNA synthesis, the second PCR was performed with plasmid DNA (20 ng) and the corresponding gene-specific primers containing T7 sequences. The dsRNA was synthesized by HiScribe T7 Quick High Yield RNA Synthesis Kit (New England BioLabs) according to the manufacturer instructions. The quality and integrity of the dsRNA was verified by standard agarose gel electrophoresis. The concentration of RNA and dsRNA was quantified by NanoDrop 2000 (Thermo Scientific). The 18–25 bp siRNA molecules corresponding to the specific RNA targets (*Bma-aldo-1*, *Bma-aldo-2*, and *gfp*) were produced by digesting the dsRNA with RNase III (Ambion); the efficiency of the reactions was verified by gel-electrophoresis of the products on a 2% agarose gel. For siRNA control treatment, dsRNA corresponding to the green fluorescent protein (*gfp*) was used.

**Table 1:** Primers for dsRNA synthesis and qRT-PCR. T7 promoter is underlined.

Gene	T7-flanked (5’ > 3’)	qRT-PCR (5’ > 3’)
***bma-aldo-1***	TAATACGACTCACTATAGGGTCGACGTTTGTATCGTCAGC TAATACGACTCACTATAGGGCAATGCTCGACCGTAACTGA	TTTGCCCGATGGTGAACAT ATCAGCGCCTTGTACGTATATGA
***bma-aldo-2***	TAATACGACTCACTATAGGGATTGGTTGATGGAGCATTGG TAATACGACTCACTATAGGGCCATGATCGTGCAGTGATTT	TCAAAATTCACAGGCAAGGTT GCATCTTTCTCCAGCTGACC
***gfp***	TAATACGACTCACTATAGGGGGTGAAGGTGATGCAACATA TAATACGACTCACTATAGGGCATCCATGCCATGTGTAATC	ACACGTGCTGAAGTCAAGTT GCTAGTTGAACGCTTCCATC
**Bm6312 (GSK-3)**		TGGGGAAAAGAAAGATGACTTG AAACGCCCAAACTGTGGATA
**Bm5931 (GP)**		TGACAATGTCTTTATTGGGAAAG CCGGAATACCAATTGATGGA
**Bm7873 (GS)**		GTTGCATACTAAGTGCAAAGCG AGTTGAGTACGTCGGAAAGCA
**Bm12811 (HK)**		TGGAATGCCAGATGAGATGA GTTTGCCAGGATTGATCGTT

### RNA interference (RNAi) treatment of *B*. *malayi* adult females

Following overnight culture, viable and motile *B*. *malayi* worms were transferred into 6 well plates in groups of 4–6 adult females per well. The siRNA treatments (final concentration 6.5 μM) were performed on female adult worms by first culturing the worms with siRNA in FBS-free culture medium (500 μL) for two hours and then with siRNA in complete medium (final volume was 5 ml), as previously described [21,23]. Treatment was continued, changing the siRNA-containing complete media every 2 days over a six-day treatment [21,23,24]. Two groups of control worms were used: parasites treated with siRNA-*gfp* and parasites with no siRNA added. Mf release was quantified by examining the number of Mf present in the media after completion of siRNA treatment and collection of adult worms. All data were analyzed using GraphPad Prism 6 and significance was determined by t-test.

### qRT-PCR and analysis of gene expression

The cDNA was prepared from three biological replicates of the treatment and control groups, each containing 2 adult female worms (*B*. *malayi*), as described above. Table 1 lists the specific primers used for the detection of *Bma-aldo-1*, *Bma-aldo-2*, Bm6312 (GSK-3), Bm5931 (GP, glycogen phosphorylase), Bm7873 (GS, glycogen synthase), and Bm12811 (HK, hexokinase) transcripts. Gene expression was estimated using the standard ‘ΔΔCt’ method. The transcript for *B*. *malayi* histone H3 was used as an internal control (reference gene) [25]. Ct values for each biological replicate were generated using three technical replicates. All data were analyzed using GraphPad Prism 6 and significance was determined using a t-test.

### DNA isolation and qPCR

DNA was extracted from individual *B*. *malayi* adult females using the QIAamp DNA Mini Kit (QIAGEN) according to the manufacturer protocol to analyze the number of *Wolbachia* per worm. *Wolbachia* numbers per individual adult parasite were quantified by qPCR using primers for a *Wolbachia* single-copy gene (*wsp*) [26]; 8–10 parasites were used for each treatment or control group. Significance was determined using a t-test.

### Protein extraction and Western blot

*B*. *malayi* adult female worms and Mf were washed in PBS three times and then homogenized in 100 μL of cold RIPA Lysis and Extraction Buffer (Thermo Scientific) plus SDS (final concentration of 5%). The lysate was then sonicated on ice for 10 minutes (30% duty cycle), followed by 10 minutes of 13,000 rpm centrifugation at 4°C. Protein concentration in the supernatant was estimated using the DC Protein Assay (Bio-Rad). Equivalent amounts of protein were electrophoresed on a 12% polyacrylamide gel followed by transfer to nitrocellulose and probing with rabbit anti-aldolase antibodies (SIGMA Aldrich) (1:2,500) overnight at 4°C. HRP-conjugated anti-rabbit secondary antibody (1:5,000) was used to detect the expression of BMA-ALDO-1 and BMA-ALDO-2 proteins. The Western blot was performed using three protein extracts analyzed in parallel. The anti-aldolase antibodies we used were generated against the conserved region of the human protein sequence (NP_000025.1), which has an overall 70% sequence similarity with aldolase-1 (39.5 kDa) and 47% similarity with aldolase-2 (42.5 kDa) of *B*. *malayi*, and no significant homology to the *Wolbachia* aldolase (wBm0097).

### MTT assay

As tetracycline-derivate antibiotics may directly reduce the activity of mitochondria [27], we used the MTT (3-(4,4-dinethylthiazol-2-yl)-2,5-diphenyltetrazolium bromide) assay to test for any effect on mitochondrial activity in nematodes after 6 days of treatment with doxycycline and in control parasites. The MTT reduction assay measures NAD(P)H flux in the cell, which is the primary product of aerobic metabolism, and thus its measurement provides an indirect measure of mitochondrial activity. Untreated control and treated adult parasites (*B*. *malayi* and *A*. *viteae*) were washed with PBS and then incubated with MTT (0.5 mg/ml) at 37°C under 5% CO_2_ for 1 hour, followed by additional wash of the parasites with PBS. The reaction product of MTT reduction (formazan) was dissolved by incubation in DMSO at 37°C for 1 hour. The soluble fractions from three to five individual worms (treated or control) were transferred into a 96-well plate and the amount of formazan present measured by the Optical Density 490 nm. Dead worms (*B*. *malayi* and *A*. *viteae*) were used as a negative control to obtain the OD that corresponds to 100% dead worms. Significance was determined using a t-test.

### Glucose and Glycogen assays

Control and *in vitro* doxycycline-treated *B*. *malayi* or *A*. *viteae* adult female worms were collected after 6 days of treatment and washed immediately with cold PBS. The samples were homogenized in distilled water on ice and the supernatants were used to measure the concentrations of both the glucose and glycogen, according to the standard protocol provided by the manufacturer (Glycogen Assay Kit, Sigma). Total protein concentrations were also estimated in all the analyzed samples using the DC Protein Assay (Bio-Rad), and they were used to normalize the measurements of glucose and glycogen levels. Results are shown in μg of glucose (or glycogen) per mg of total protein in 1 ml of soluble worm extract. Three to five biological replicates were collected for control and antibiotic treated parasites on day 6 and two technical replicates were performed for each measurement. Significance was determined using a t-test.

### TUNEL assay

Treated and control *B*. *malayi* adult female worms were fixed in 4% formaldehyde (Sigma) in PBS for 20 minutes at room temperature and stored at 4°C until use. The adult female worms were cut into several fragments, releasing embryos, and the fixed segments of worms and embryos were permeablized in PBS with 0.1% Triton-X100 (PBST) for 30 minutes. Extracted embryos were treated with RNAse A (Sigma) at 100 μg/ml overnight at 4°C in PBST. The following day, an additional permeabilization step was performed using DNAse-free proteinase K (QIAGEN) for 30 minutes at 37°C at a concentration of 20 μg/ml in 10 mM Tris HCl (pH 7.5). After washing in PBST, TUNEL staining (37°C for 1 hour) was performed following the manufacturer’s protocol (In Situ Cell Death Detection Kit, Fluorescein, Roche). The embryos were mounted in Vectashield medium containing propidium iodide to visualize DNA (host nuclei and *Wolbachia*) and the proportion of embryos with apoptotic nuclei was quantified 24 hours later using a Zeiss 510 Meta confocal microscope.

### Microscopy

For the visualization of aldolase localization, *B*. *malayi* adult females were fixed and permeabilized using 4% formaldehyde in PBS (Sigma) with 0.1% Triton-X100 (PBST) for 20 minutes at room temperature. During fixation, worms were cut several times to improve the penetration of the reagents. Samples were then washed three times in PBST and treated with RNase A (100 μg/mL) overnight at 4°C. The following day, samples were washed in PBS and blocked for 15 minutes in PBS containing 1% BSA. Next, the samples were incubated overnight at 4°C with rabbit anti-aldolase antibodies (1:500) in PBS containing 1% BSA. After washing, samples were incubated at room temperature for 1 hour with FITC conjugated anti-rabbit secondary antibodies (1:500) in PBS containing 1% BSA. After washing, the samples were mounted in Vectashield medium containing propidium iodide to visualize DNA (host nuclei and *Wolbachia*) and were viewed using a Zeiss 510 Meta confocal microscope.

Transmission electron microscopy (TEM) was performed with worms fixed with 2.5% glutaraldehyde in PBS for 2 hours, and post fixed with 1% OsO4 for 1 hour. During fixation, worms were cut into ∼5 mm pieces. The samples were then dehydrated using an increasing series of ethanol concentrations (50–100%) and acetone. Samples were embedded in Agar 100 epoxy resin and cut into ultrathin sections (60–70 nm). The sections were contrasted with uranyl acetate and lead citrate and observed using an FEI Tecnai 12 Spirit electron microscope.

For immuno-transmission electron microscopy analyses, worms were fixed with 4% paraformaldehyde and 0.25% glutaraldehyde in PBS for 4 hours at 4°C. During fixation, worms were cut into ∼5 mm pieces. After fixation, samples were washed three times in PBS and dehydrated using a series of increasing ethanol concentrations (50–100%). Samples were then embedded in Lowicryl resin (Gold) and cut into ultrathin sections (60–70 nm). The sections were pre-incubated with 1% BSA in PBS before overnight incubation with anti-aldolase antibodies (1:50) in PBS containing 1% BSA. The sections were finally incubated with secondary antibodies (1:50) conjugated with gold particles (15 nm) and contrasted as described above for the analysis using a FEI Tecnai 12 Spirit electron microscope.

## Results

### Characterization of the two *B*. *malayi* fructose-1,6-bisphosphate aldolase enzymes

**Fig 1:**
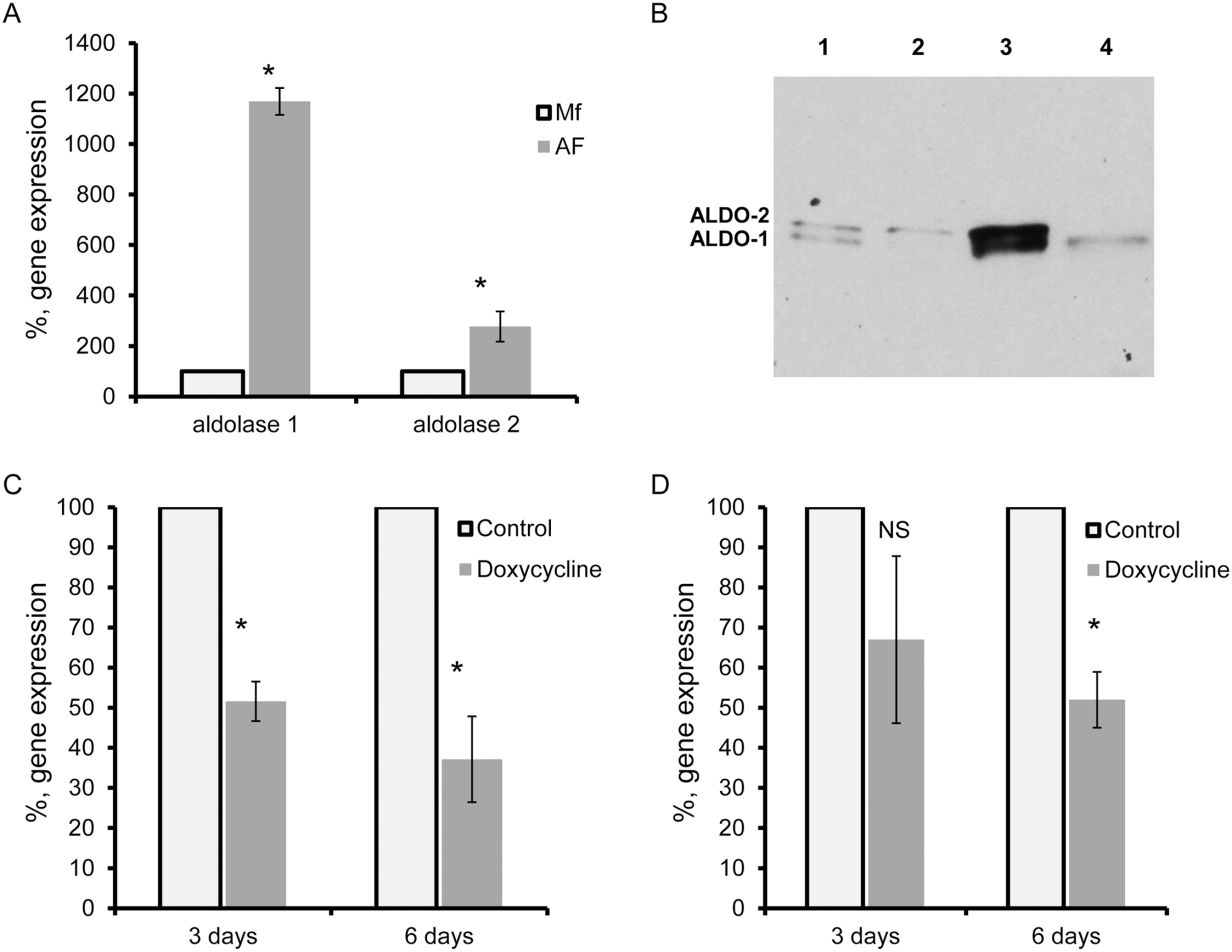
Expression of aldolase in *B*. *malayi*. (A) *Bma-aldolase-1* and *Bma-aldolase-2* are overexpressed in adult female worms as compared to microfilaria (Mf). Relative gene expression between females and Mf was calculated using the ΔΔCt method. The median value of the Mf group was set to 100% and the increase in expression in the female worms was calculated as a percentage of the control. (B) Western blot analysis shows the expression of BMA-ALDO-1 and BMA-ALDO-2 proteins in Mf (lane 1), in 6-day doxycycline-treated Mf (lane 2), in adult females (lane 3), and in 6-day doxycycline-treated adult female worms (lane 4). Reduction of *Bma-aldolase-2* (C) and *Bma-aldolase-1* (D) expression in doxycycline (3- and 6-day, grey bars) treated female worms as compared to control untreated worm samples (transparent bars). The median value of the control group was set to 100% and the reduction in expression in the treated groups was calculated as a percentage of the control. *: *p* < 0.001, NS: not significant.

The *B*. *malayi* genome contains two genes encoding putative aldolase isozymes: BMA-ALDO-1 (Bm5580), and BMA-ALDO-2 (Bm3135), which are 56% similar at the amino acid level. Using gene-specific primers (Table 1), we analyzed by qRT-PCR the expression of both aldolases in fertile adult female and Mf. Of all the developmental stages of *B*. *malayi*, Mf contains the smallest number of *w*Bm while the adult female worms carry the maximum number of bacteria [26]. The expression levels of *Bma-aldo-1* and *Bma-aldo-2* transcripts were significantly higher (11.7 and 2.7 times, respectively) in adult female worms when compared to Mf (Fig 1A). Western blot analyses using monospecific antibodies raised against a conservative domain of the human and parasite aldolase proteins confirmed that both proteins, BMA-ALDO-1 (39.5 kDa) and BMA-ALDO-2 (42.5 kDa), are also highly expressed in *B*. *malayi* adult females as compared to Mf (Fig 1B).

To determine whether aldolase expression levels are affected by *w*Bm fitness, transcript levels of both enzymes were measured in adult female worms in which *Wolbachia* were eliminated by *in vitro* doxycycline treatment. There was a significant decrease of *Bm-aldo-2* expression (approximately 50%) in the adult parasites after 3-days of treatment and it was further reduced (>63%) after 6 days of treatment when compared to the control group (Fig 1C). The expression of *Bm-aldo-1* in adult female worms did not change significantly after 3-days of treatment as compared to control samples but a 48% decrease of *Bm-aldo-1* transcripts was observed in worms after 6-days of treatment when compared to the control group (Fig 1D). Notably, the expression of both proteins was reduced in the 6-day doxycycline treated adult female worms as compared to the untreated females (Fig 1B, Lanes 3 and 4).

### Inhibition of aldolase-2 has a dramatic effect on both the parasite and its symbiont

**Fig 2:**
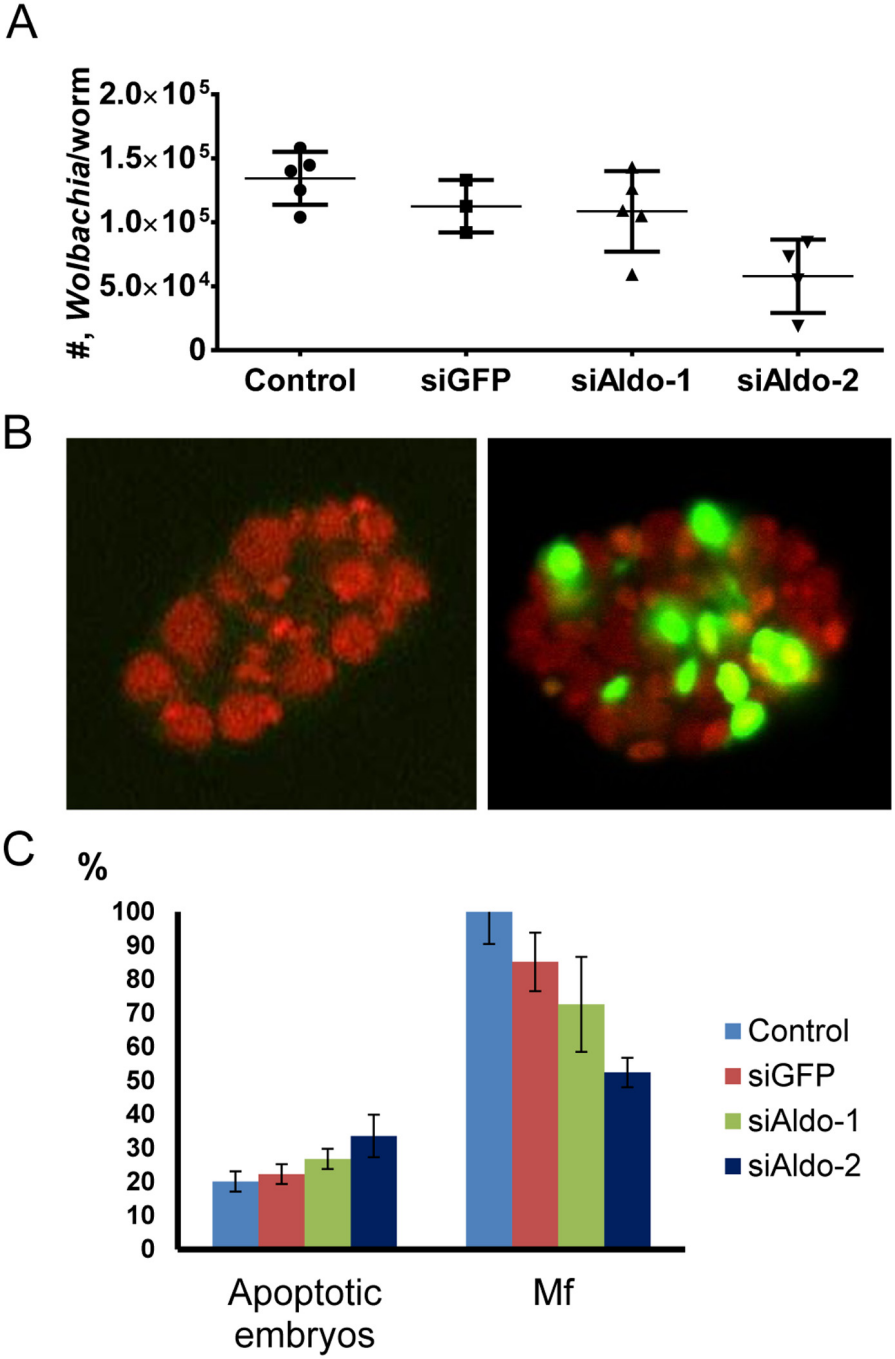
Silencing of *Bma-aldolase-2* decreases *Wolbachia* load in adult female worms and induces apoptosis in developing embryos. (A) qPCR analysis of the number (#) of *wsp* copies per adult female worm at the end of the RNAi experiments. Significant reduction of *Wolbachia* load was only detected in females treated with siRNA specifically targeting *Bma-aldolase-2* (siALDO-2) as compared to RNAi-control (worms were treated with siRNA-GFP; *p* < 0.01) or untreated worms (*p* < 0.01). (B) TUNEL assay for developing embryos extracted from control (left) or treated with siRNA specific to *Bma-aldolase-2* (right). Green represents apoptotic nuclei and all nuclei were also co-stained with a DNA-specific dye (propidium iodide, red), magnification 60X. (C) Number of apoptotic embryos extracted from females and number of microfilaria (Mf) released from control females and females after treatment with siRNAs. The increase in the proportion of apoptotic embryos in worms treated with siRNA specifically targeting *Bma-aldolase-2* (siALDO-2) was significant as compared to the RNAi-control (*p* < 0.05) or untreated (*p* < 0.05) worms. The number of Mf released by worms treated with siRNA specifically targeting *Bma-aldolase-2* (siALDO-2) was about 50% of those released by RNAi-control (*p* < 0.05) or untreated (*p* < 0.05) worms.

To further elucidate the importance of aldolase in the *w*Bm–*B*. *malayi* relationship, we performed a functional analysis of aldolase in *B*. *malayi* using short interfering RNA (siRNA) specifically designed to silence either *Bma-aldo-1* or *Bma-aldo-2*. The silencing of *Bma-aldo-2* significantly decreased *w*Bm (a decrease of 55%; p<0.01) (Fig 2A) and induced apoptosis in ~34% (p<0.05) of the developing embryos (Fig 2B and 2C) when compared to control worms treated with siRNA-GFP. The number of Mf released from the siRNA-aldolase-2-treated female worms was also significantly reduced (by 33%; p<0.05) as compared to the siRNA control group (Fig 2C). In contrast, the silencing of *Bma-aldo-1* had no significant effect on the number of *Wolbachia* in the treated worms, and did not alter either the motility of the female worms or embryogenesis (Fig 2).

### *B*. *malayi* aldolase is associated with both *Wolbachia* and host glycogen

**Fig 3:**
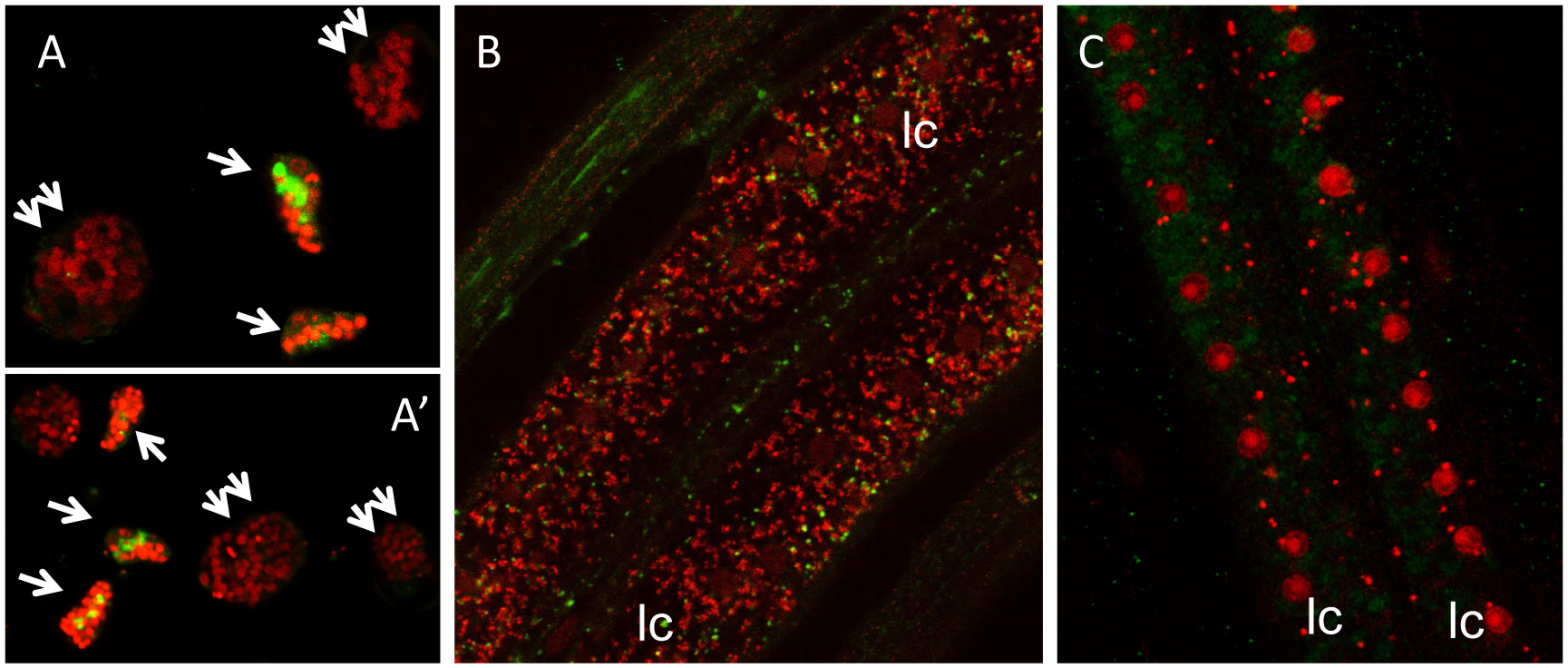
Aldolase in *B*. *malayi* is predominantly found in the lateral cord of adult worms and in early embryos. Aldolase (green FITC staining with antibodies) is expressed in early embryos (arrow) and, to a much lesser degree, in developing embryos (double arrow) (A, A’), and in the lateral chord of adult females (B). Its expression is reduced in the lateral chord of doxycycline-treated adult female worms (C). Red represents a propidium iodide staining for DNA (large spots represent nuclei, small dots, *Wolbachia*), lc: lateral cord. Magnification 60x.

**Fig 4:**
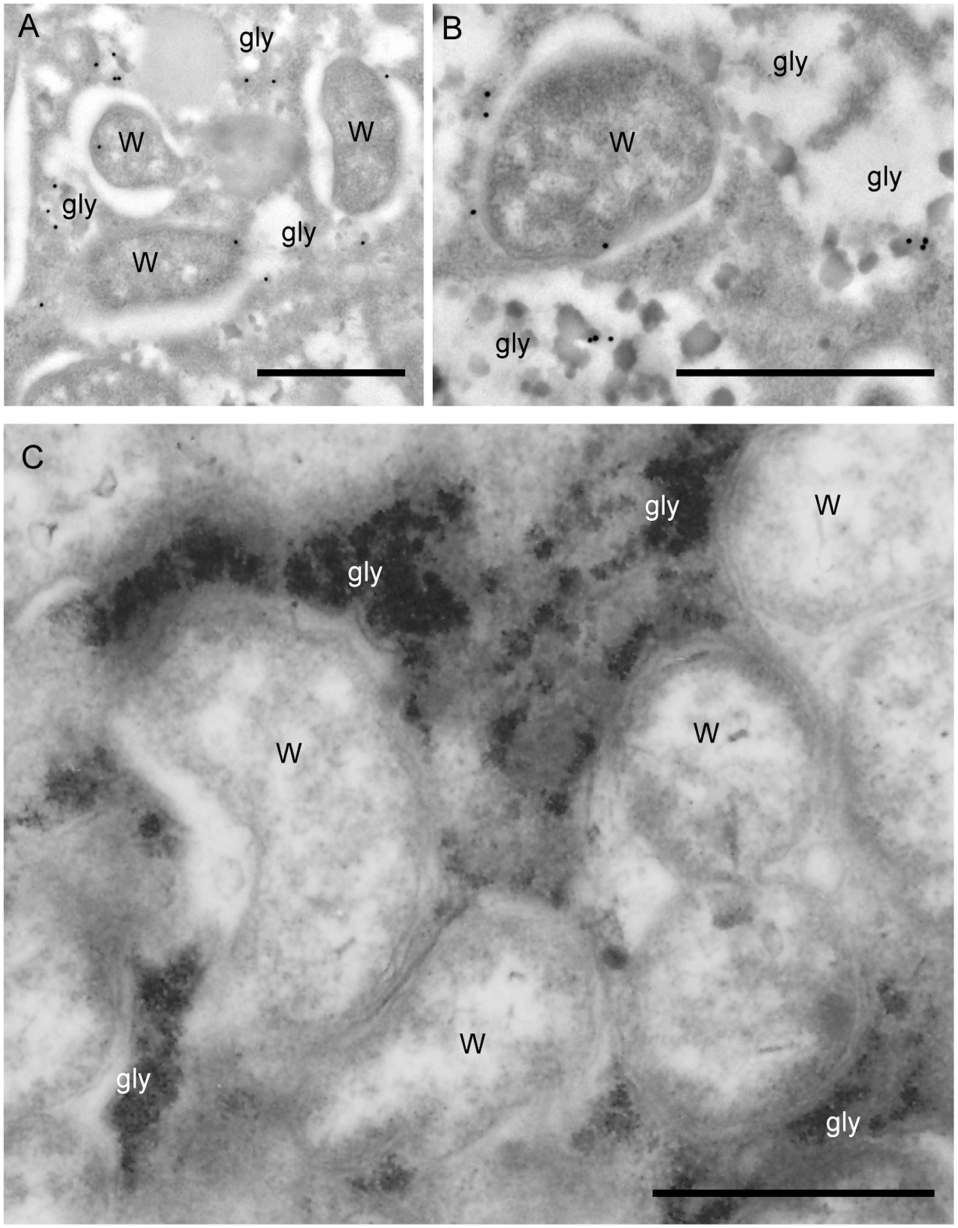
Co-localization of *Wolbachia*, aldolase and glycogen in the lateral cord of *B*. *malayi*. (A) and (B), Immunogold labelling showing the localization of aldolase on *Wolbachia* (W) surface and within glycogen (gly). (C) Transmission electron micrograph showing *Wolbachia* (W) embedded within the granules of glycogen (gly) in the cytoplasm of the lateral chord. Bar = 1 μm.

Using confocal microscopy and anti-human aldolase antibodies that can detect both *B*. *malayi* proteins, we found that the aldolase enzymes were strongly expressed in the lateral cord of the female worms, where most of the bacteria reside (Fig 3). Moreover, we found aldolase to be expressed at a high level in the reproductive tract, specifically in the early embryos, while the expression gradually decreased in the developed embryos (Fig 3A and 3A’). The expression of aldolase in the lateral chord of doxycycline treated worms was reduced (Fig 3B and 3C). In our previous study we have shown the co-localization of aldolase with *w*Bm in the cytoplasm of the lateral cord of *B*. *malayi* female worms [19]. In the current study we observed that the enzyme is localized on the bacterial surface, but not in the matrix of the bacteria (Fig 4A and 4B). Furthermore, aldolase was found to be associated with glycogen in the cytoplasm of lateral cord cells (Fig 4A and 4B). Notably, *w*Bm are also embedded within the granules of glycogen present in the hypodermal cord of adult female worms (Fig 4C, S1 Fig). The association of both aldolase and *w*Bm with glycogen prompted us to further explore the possibility that bacteria may regulate the components of the host glycolytic pathway by affecting glycogen and/or glucose utilization.

### *Wolbachia* regulate glycogen metabolism in *B*. *malayi* female worms

**Fig 5:**
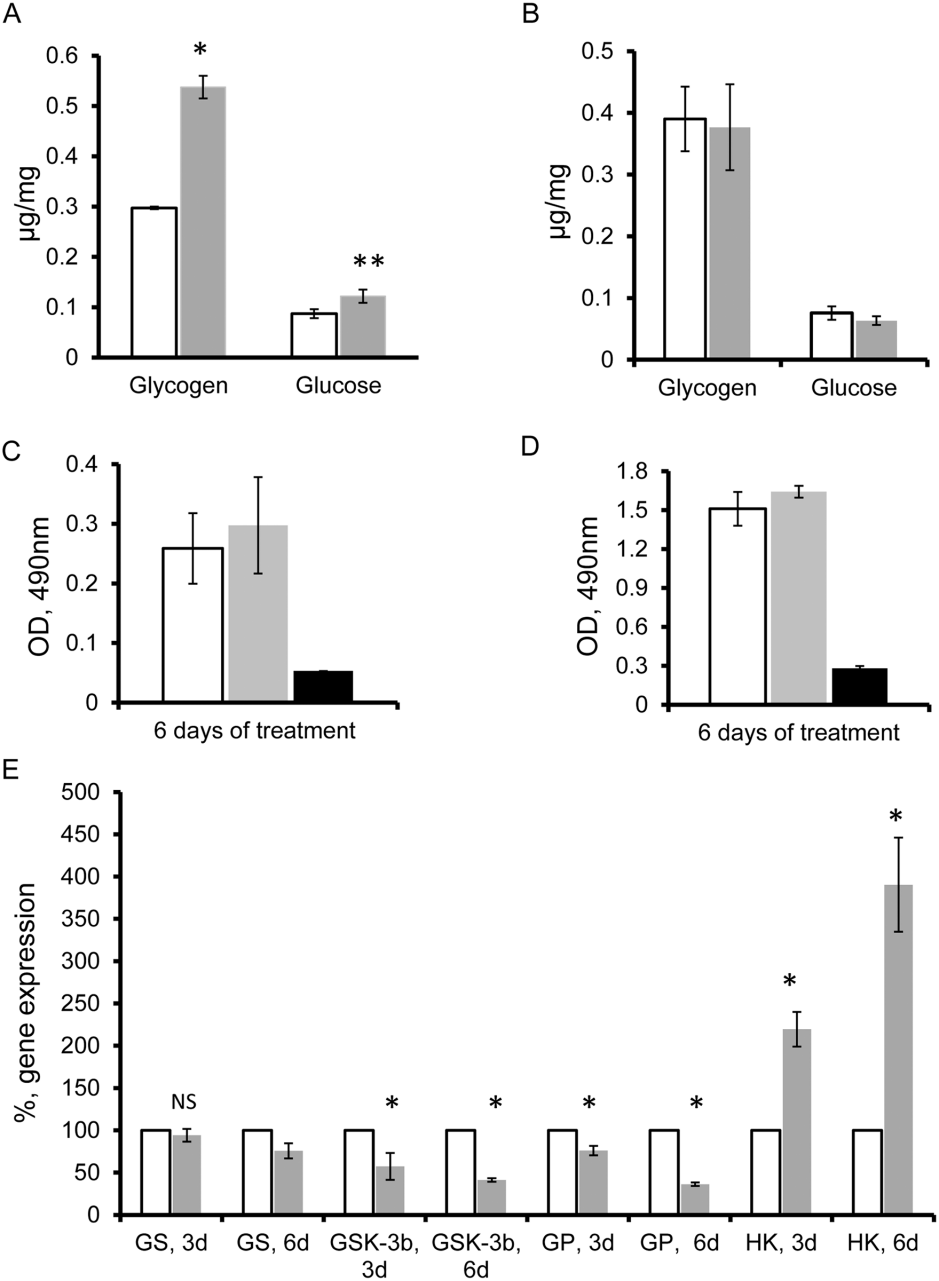
Decreased fitness of *Wolbachia* leads to increased glucose and glycogen amounts in the *B*. *malayi* female worms, and regulates the expression of genes that are part of the glycogen metabolic pathway. Increase in glucose and glycogen levels (A) observed in 6-day doxycycline-treated *B*. *malayi* female worms (grey bars) as compared to control worms (transparent bars). The Y axis shows μg of glucose (or glycogen) per mg of total protein in 1 ml of soluble worm extract (*: *p* < 0.01, and **: *p* = 0.02 between control and treated samples). (B) Similar glucose and glycogen levels are found in 6-day doxycycline-treated *A*. *viteae* (*Wolbachia*-free filariae) female worms (grey bars) and in control *A*. *viteae* worms (transparent bars). (C) and (D) Optical Density levels obtained from MTT assay performed on 6-day doxycycline-treated (grey bars) and control (transparent bar) *B*. *malayi* (C) and *A*. *viteae* (D). Black bars (C and D) represent OD levels measured in dead worms. (E) Percent of relative gene expression of the following genes: glycogen synthase (GS), glycogen synthase phosphatase-3 (GSK-3), glycogen phosphorylase (GP), and hexokinase (HK) in 3-day doxycycline-treated (3d) and 6-day doxycycline-treated (6d) *B*. *malayi* female worms (grey bars) and in control samples (transparent bars). The median value of the control group was set to 100% and the reduction or increase in the expression levels of these genes in the treated groups was calculated as a percentage of the control. *: *p* < 0.001, as determined by comparing the values between doxycycline-treated and control worms for each experimental treatment group. NS: not significant.

To test the hypothesis that the presence of *w*Bm may also regulate glycogen metabolism in the host lateral cord, the levels of glucose and glycogen in the adult *B*. *malayi* females were examined in control and doxycycline-treated worms. First, we determined whether the amount of glucose and its polymer (glycogen) within the female worms depended on bacterial fitness. Glucose and glycogen levels were quantified in 6-day doxycycline-treated parasites using an enzymatic assay, as described in Materials and Methods. The amounts of both glucose and glycogen were significantly higher in doxycycline-treated female worms than in the control worms (p = 0.02 and p<0.01, respectively; Fig 5A). To demonstrate that this increase was associated with the decreased number of *Wolbachia* and not to secondary effects of drug treatment, glucose and glycogen levels were measured in *A*. *viteae* cultured in the presence and absence of doxycycline. *A*. *viteae* is a rodent filarial parasite that lacks a *Wolbachia* endosymbiont. Female *A*. *viteae* worms treated with antibiotic for six days had similar amounts of both glucose and glycogen deposits when compared to DMSO-treated (control) worms (Fig 5B).

Tetracycline-derivate antibiotics may also suppress mitochondrial activity in eukaryotic cells [27], and therefore may directly affect glucose metabolism in mitochondria. To ensure that the effect observed with antibiotic treatment was *Wolbachia*-specific and did not reduce mitochondria activity in the filarial nematodes by inhibiting the normal process of pyruvate uptake by mitochondria, we performed an MTT reduction assay on parasites (*B*. *malayi* and *A*. *viteae*) after 6 days of doxycycline treatment. The MTT reduction assay estimates indirectly the mitochondrial activity in cells by measuring the primary product of aerobic metabolism (NAD(P)H). There were no significant differences in NAD(P)H levels between the doxycycline-treated and untreated *B*. *malayi* or *A*. *viteae* parasites (Fig 5C and 5D), implying that the changes in glucose and glycogen metabolism we found are associated to the *w*Bm and *B*. *malayi* interaction, and bacterial fitness.

To further characterize the glycogen metabolic pathway in *B*. *malayi* and its possible association with *w*Bm, we tested the expression level of glycogen synthase (GS, Bm7873), an enzyme involved in the polymerization of glucose. While glycogen synthase activity was not significantly affected in the 3-day doxycycline-treated worms, it was decreased by 31.5% in the parasites after the 6-day treatment (Fig 5E). Transcript levels of glycogen synthase kinase 3b (GSK-3b, Bm6312), an inhibitor of GS protein, however, decreased by 31% after 3 days and by 57% after 6 days of treatment, as compared to the control worms (Fig 5E). To assess glycogen utilization we also evaluated the gene expression of glycogen phosphorylase (GP, Bm5931), an enzyme that catalyzes the breakdown of glycogen to glucose. A 25% and 64% decrease in GP gene expression was present in the 3- and 6-day doxycycline-treated worms, respectively, as compared to control worms (Fig 5E).

The glycogen metabolic pathway is regulated by the amount of glucose available [28]. Generally an increase of glucose concentration in eukaryotic cells results in downregulation of glycogen breakdown and an increase in glycogen synthesis. A higher cytoplasmic concentration of glucose provides increased cytoplasmic levels of glucose-6-phospate (G-6-P), the product of hexokinase (HK). Glucose-6-phospate negatively regulates GSK-3b, which in turn up-regulates glycogen synthase activity. This mechanism changes the direction of utilization of glucose from glycolysis to glycogen synthesis (storage). Notably, the transcript level of HK in doxycycline-treated *B*. *malayi* females was >2 and 4 times higher than in the control worms after 3- and 6-day treatment with doxycycline, respectively (Fig 5E). The combination of these data supports the notion that reduction of *Wolbachia* fitness by antibiotic treatment may have decreased bacterial demand on glucose and as a result the filarial host has decreased the metabolism of glucose and glycogen.

## Discussion

**Fig 6:**
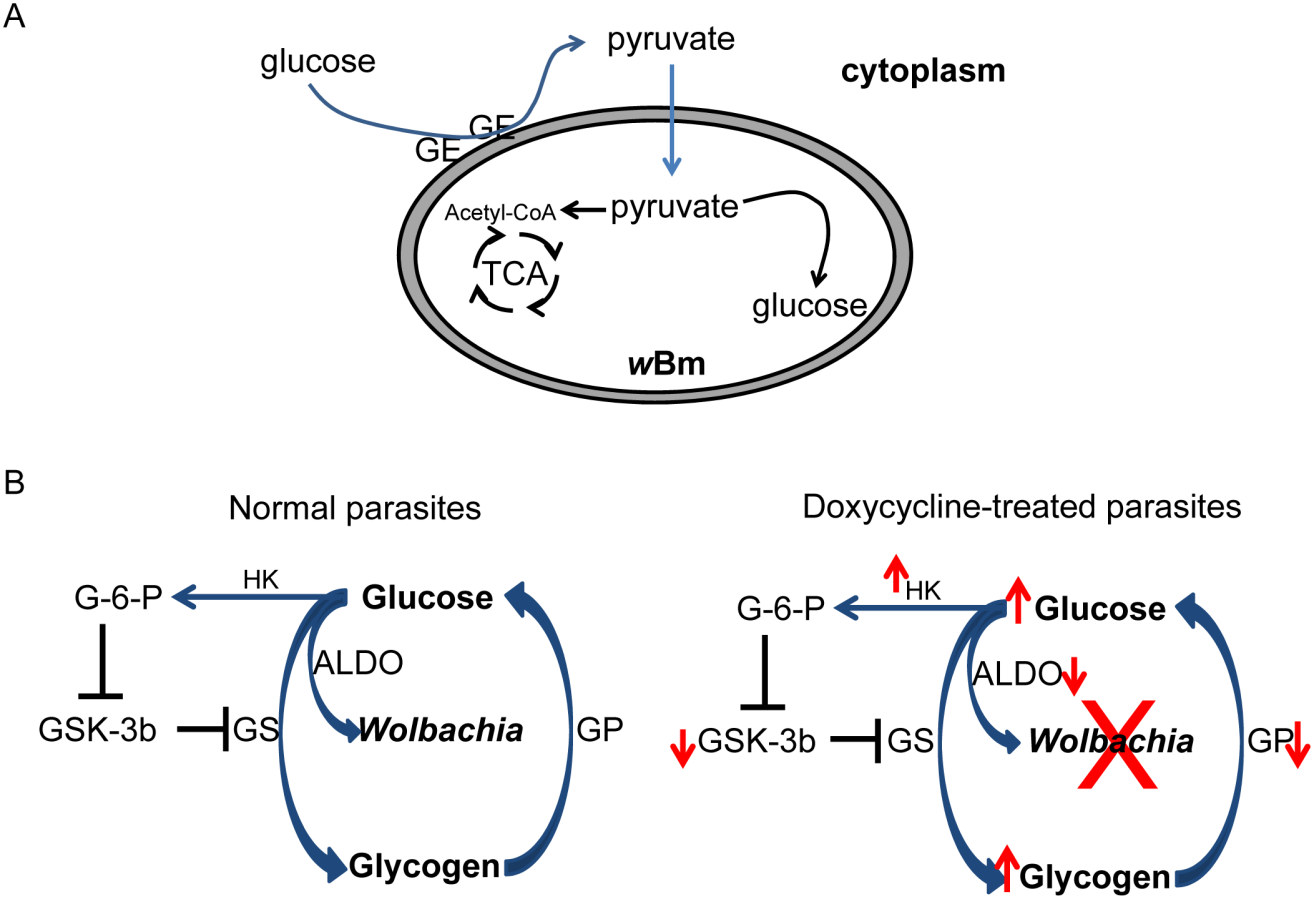
Schematic representation of the role *w*Bm may play in regulating the metabolism of glucose and glycogen in *B*. *malayi*. (A) *w*Bm require pyruvate for intracellular glucose and energy production. Parasite glycolytic enzymes (GE) are clustered to the bacterial surface in a complex with wBm00432, which can support the conversion of the intracellular glucose into pyruvate. Pyruvate is then utilized by the bacteria either for energy production or gluconeogenesis. (B) Bacteria change the expression of host genes that are involved in the glycogen metabolic pathway. A decline in bacterial fitness due to antibiotic treatment increases glucose levels and decreases utilization of glycogen, resulting in an increase in glycogen stores as well. Abbreviations: TCA: tricarboxylic acid cycle, GS: glycogen synthase, GP: glycogen phosphorylase, GSK-3: glycogen synthase phosphatase-3, HK: hexokinase, G-6-P: glucose-6-phosphate.

Obligatory endosymbiotic bacteria living in the cytoplasm of eukaryotic cells rely on the host’s intracellular environment as the primary source for certain metabolites, energy, nutrients, amino acids, and/or fatty acids [2,16,22,29,30]. Studies of *Wolbachia* in filarial worms have revealed specific functional and structural associations with the host organelles, such as the endoplasmic reticulum, mitochondria, and intracellular vesicles [31,32]. Analysis of the *w*Bm genome indicates that the bacteria are missing two key enzymes in the glycolytic pathway, 6-phosphofructokinase and pyruvate kinase, and thus are not able to directly convert glucose into pyruvate *de novo* [17,18]. However, all the components of the gluconeogenesis pathway, which utilizes pyruvate as its primary starting material, are present in the bacterial genome (Fig 6) [13]. In the present study, we present evidence supporting a new role in the symbiotic relationship for one of the host glycolytic enzymes, fructose-1,6-bisphosphate aldolase, found to be associated with a *Wolbachia* surface protein [19]. Fructose-1,6-bisphosphate aldolase is a glycolytic enzyme that catalyzes the conversion of fructose-1,6-biphosphate into glyceraldehyde 3-phosphate and dihydroxyacetone phosphate during glycolysis—the process by which glucose is catabolized into pyruvate [33]. The genome of *B*. *malayi* encodes two aldolase isoforms (BMA-ALDO-1 and -2) and both appeared to be down-regulated in *B*. *malayi* worms that were partially depleted of *w*Bm. However, RNAi mediated knockdown of BMA-ALDO-2 expression had a major effect on *Wolbachia*’s fitness when compared to BMA-ALDO-1, suggesting that BMA-ALDO-2 is probably more essential for *w*Bm and *B*. *malayi* maintenance of symbiosis. Aldolase-2 may thus have dual roles: in addition to its central role in glycolysis for the filarial host and possibly also during embryogenesis, where it also plays a role in the *Brugia malayi-w*Bm symbiotic interaction. Aldolase-2 in the free living *C*. *elegans* nematode is specifically expressed during embryogenesis and early larval stages [34], and the silencing of *Ce-aldo-2* resulted in embryonic mortality (F01F1.12, RNAi.org). Its second role in the *Wolbachia* containing filarial worms is evidenced by showing that reduction in BMA-ALDO-2 expression also decreased the *Wolbachia* load. The effect on embryonic lethality might be a combination of both roles.

The co-localization of aldolase and *Wolbachia* within intracellular glycogen (a major source of glucose) suggested that *Wolbachia* may utilize the parasite’s glycogen deposits as an energy source. We found that the elimination of *w*Bm from *B*. *malayi* adult female worms by doxycycline increased the amounts of available glucose and its intracellular polymer, glycogen. We hypothesize that this could be due to the decreased utilization of glycogen and/or a reduced indirect demand for glucose metabolism by the *Wolbachia* (Fig 6). This hypothesis is supported by two additional observations. First, we eliminated the possibility that doxycycline, a tetracycline-related antibiotic, might have had a strong effect on the mitochondria within the worms, and thus indirectly caused their decreased activity leading to a disruption of glucose/glycogen metabolism in the doxycycline-treated parasites. Doxycycline treatment did not reduce mitochondrial activity in either *B*. *malayi* or *A*. *viteae*, as assessed by the MTT reduction assay. Second, we excluded a possible direct effect of the antibiotic treatment on increasing glucose or glycogen deposit by performing the same experiment on the *Wolbachia*-free worms *A*. *viteae*. The antibiotic treatment did not change the amount of glucose or glycogen in treated female *A*. *viteae* parasites when compared to control worms. These data, when taken together, suggest that the alterations in glycogen levels that we observed in doxycycline treated *B*. *malayi* were a direct result of depletion of *Wolbachia* by the antibiotic treatment.

In previous studies we found that the *B*. *malayi* aldolase and several other glycolytic enzymes are associated with the *Wolbachia* surface via a complex with the wBm0432 outer membrane protein [19]. Here, we were able to determine that aldolase is not only localized to the surface of *Wolbachia* but that it is also associated with clusters of intracellular host glycogen. Based upon these findings, we propose that *Wolbachia* recruits *B*. *malayi* glycolytic enzymes to its surface to access pyruvate and/or other metabolites that provide an energy source, and that it subsequently regulates glucose and glycogen metabolism as needed.

Glycogen utilization is decreased through the suppression of glycogen phosphorylase (GP), an enzyme involved in the breakdown of glycogen [35]. Microarray analysis of gene expression during depletion of *Wolbachia* in *Litomosoides sigmodontis*, a rodent filarial nematode, showed a reduction in GSK-3 expression at day 6 of treatment [36]. Glycogen synthase is regulated through the GSK-3 kinase activity [37,38]. Although the gene expression of glycogen synthase did not increase in the doxycycline-treated *B*. *malayi*, the activity of the enzyme might be increased through significant suppression of expression of GSK-3, a negative regulator of GS activity. It has been shown that GS activity is stimulated by glucose 6 phosphate (G-6-P), the product of phosphorylation of glucose by hexokinase. This effect is mediated by the allosteric inhibition of GSK-3 by G-6-P. We observed that hexokinase transcript levels were up-regulated in doxycycline-treated *B*. *malayi* female adult worms. Thus, it is possible that this increase of hexokinase expression leads to an increase of G-6-P and subsequent inhibition of GSK-3, resulting in a higher GS activity and glycogen accumulation.

Intracellular pathogens have also been shown to influence host glycogen synthesis in other biological systems. For example, *C*hlamydia encodes enzymes for glycogen metabolism, mutations of which increases glycogen deposition in epithelial cells [39]. Since *Wolbachia* lack the genes necessary for glucose catabolism, we propose that their preferential association with the host glycolytic enzymes allows them to acquire pyruvate, the end product of host glycolysis. Decreasing fitness of *Wolbachia* by antibiotic treatment reduces the demand by the endosymbiont for pyruvate, thereby reducing glycolytic activity in the filarial host. We hypothesize that *Wolbachia* modifies parasite glycolytic enzyme activity and, as a result, the bacteria fundamentally alter the metabolic demands from the host intracellular environment (Fig 6).

It is tempting to speculate that BMA-ALDO-2 could become a potential novel drug target for anti-filarial treatment via the reduction of both nematode host and *Wolbachia* fitness. Aldolase has been considered as a valid drug target for the elimination of other intracellular pathogens (reviewed in [40]). Aldolase of the human malarial parasite (*Plasmodium falciparum*) is a potential target for antimalarial structural drug design; the erythrocytic stages of the parasite, as well as invasion of new red blood cells by the merozoites are dependent on glycolysis [41,42]. Glycolysis deregulation is also present in other non-infectious diseases. It has been observed that the high activity of cancer cells and their growth is driven by a high glucose metabolism (also known as the “Warburg effect”) [43]. Consequently, as part of anti-cancer research several small molecule inhibitors of glycolysis (such as 3-Br-pyruvate) have been developed to decrease the metabolism of glucose that could be crucial for proliferation of cancer cells [44–46]. Other glycolytic enzymes such as enolase are also considered as valid drug targets for anti-trypanosome treatment due to high metabolic demand in the parasites [47]. Such newly available drugs that target glycolysis could be tested in future studies in the various filarial screening systems [48] to demonstrate their possible effects on the fitness of both the symbiont and its filarial host.

## Supporting Information

S1 Fig *Wolbachia* are embedded within glycogen in the lateral cord of *B*. *malayi*. (A), Transmission electron micrograph showing *Wolbachia* (W) embedded within the granules of unconverted glycogen (gly) in the cytoplasm of the lateral chord. Bar = 1 μm.
(TIF)
