# Supplementary figures and images for: Glucose and Glycogen Metabolism in Brugia malayi Is Associated with Wolbachia Symbiont Fitness
Source: PLoS One. 2016 Apr 14;11(4):e0153812. doi: 10.1371/journal.pone.0153812 (PMC4831766; doi:10.1371/journal.pone.0153812)

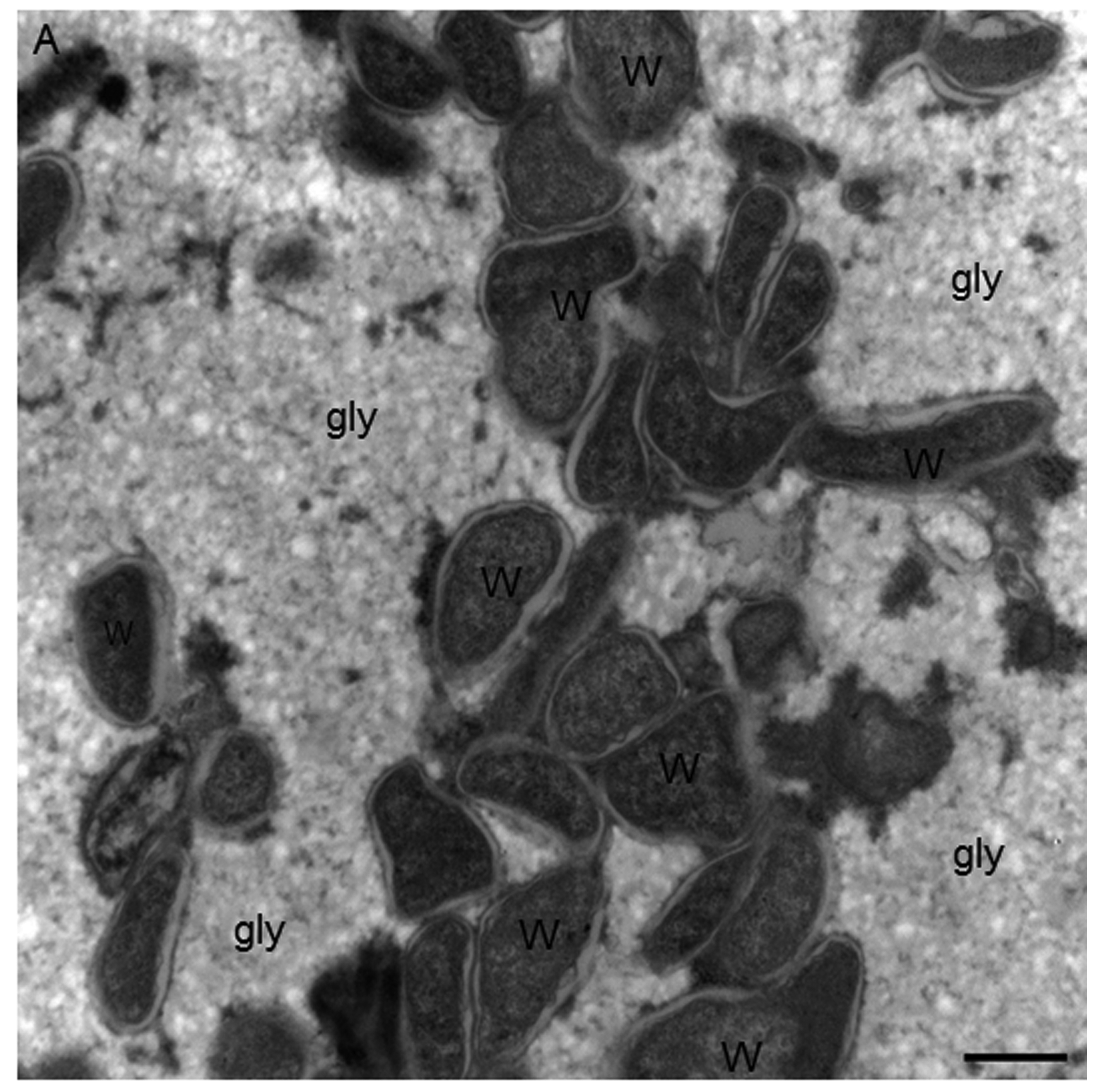

Supplement: S1 Fig — (A), Transmission electron micrograph showing Wolbachia (W) embedded within the granules of unconverted glycogen (gly) in the cytoplasm of the lateral chord. Bar = 1 μm. (TIF) [file pone.0153812.s001.tif]
